# Sleep and Diabetes

**DOI:** 10.1155/2010/759509

**Published:** 2010-03-09

**Authors:** Swetha Bopparaju, Salim Surani

**Affiliations:** ^1^Section of Pulmonary, Critical and Sleep Medicine, Department of Medicine, Baylor College of Medicine, Corpus Christi, TX 78413, USA; ^2^Baylor College of Medicine, Texas A&M University, 613 Elizabeth Street, Suite 813, Corpus Christi, Houston, TX 78413, USA

## Abstract

Sleep apnea is clinically recognized as a heterogeneous group of disorders characterized by recurrent apnea and/or hypopnea. Its prevalence ranges from 4% to 24%. It has been implicated as an independent risk factor for several conditions such as hypertension, stroke, arrhythmia, and myocardial infarction. Recently data has been emerging which suggests an independent association of obstructive sleep apnea with several components of the metabolic syndrome, particularly insulin resistance and abnormalities in lipid metabolism. We hereby review the salient features of the association between sleep and diabetes.

## 1. Introduction

Sleep apnea is clinically recognized as a heterogeneous group of disorders characterized by recurrent apneas (complete cessation of breathing) and/or hypopnea (decrease in airflow with desaturation of 4%) [1]. It is estimated that obstructive sleep apnea may affect at least 2%–4% of the general population, with prevalence estimates that may be much higher based on demographic variables such as age, sex, and body mass index (BMI) [2]. The apnea-hypopnea index (AHI) was defined as the total number of obstructive apneas (cessation of airflow for at least 10 seconds) and hypopneas (decrease of the airflow signal amplitude by at least 50% accompanied by oxyhemoglobin desaturation of at least 4% or by an arousal) per hour of sleep [3]. An AHI of 10 or higher and excessive day-time somnolence has also been used to define obstructive sleep apnea (OSA), finding similar figures in a large cohort of male subjects [4]. Data from Sleep in America poll by The National Sleep Foundation showed that approximately 25% of adults and 57% of obese individuals are at high risk for obstructive sleep apnea (OSA) [5]. Sleep apnea has been implicated as an independent risk factor for hypertension, myocardial infarction, and stroke [6]. 

Diabetes Mellitus is a well-established risk factor for cardiovascular disease. Data from the Third National Health and Nutrition Examination survey indicate that 5.1% of adults in the United States have physician diagnosed Diabetes Mellitus and an additional 2.7% meet the criteria of diabetes but remain undiagnosed [7]. Given the pandemic of obesity, the prevalence of Type 2 Diabetes Mellitus(DM) and metabolic syndrome has also increased dramatically over the last decade [8–12]. DM affects approximately 6% of adults in the United States [13]. There have been several studies which have suggested an independent association of obstructive sleep apnea with several components of metabolic syndrome, particularly insulin resistance and abnormalities in lipid metabolism [14–17]. 

There has been data that long-term intermittent hypoxia and sleep fragmentation increase sympathetic activity, which in turn lead to disorders of glucose metabolism [18]. Alternatively some studies have suggested that insulin resistance and chronic hypoxemia may in turn lead to development of sleep apnea syndrome [19]. 

The Sleep Heart Health Study found that subjects with DM had increased sleep disordered breathing and more severe hypoxemia [20]. Factors influencing and increasing the risk of sleep apnea include male sex [1, 21, 22], obesity [23, 24], age [4, 25], and race [25, 26]. Studies done to look at the association of DM as an etiologic factor for sleep apnea have suggested that autonomic neuropathy may be a responsible for dysfunction of central respiratory control of the diaphragm and decreased upper airway tone. Somers et al. found that sleep disturbance negatively affects glucose metabolism and endocrine function. After six nights of four hour sleep, sympathetic nervous activity has increased (*P* < .02) [27]. Findings from epidemiological studies indicate that sleep apnea is independently associated with hypertension [28] and cardiovascular disease [29]. A growing body of literature suggests that sleep apnea is associated with fasting hyperglycemia, insulin resistance, and DM [30]. 

Several factors have been associated with diabetes in sleep from obesity to altered glucose metabolism. Insulin resistance, glucose intolerance, REM sleep and diabetes, and the effect of sleep apnea treatment on diabetes have been implicated as causation factors. We will try to summarize this complex subject in as simple a form as possible. 

## 2. Obesity, OSA, and Diabetes

Obesity is generally regarded as a risk factor for both OSA and Insulin resistance [31, 32]. During the past 20 years, the prevalence of obesity and DM in the United States has increased consistently [33]. Obesity, in particular central obesity, is the strongest risk factor for sleep apnea [34–41]. OSA is prevalent particularly among middle-aged, obese men, although its existence in women and lean individuals is being increasingly recognized. 

There is an alarming rise in the prevalence of DM that may be largely attributed to the epidemic of obesity [42]. The strongest risk factor for both DM and OSA is obesity with a high visceral (central) fat distribution [43, 44]. Approximately two thirds of all patients with OSA are obese, and the effect of obesity as a predictor of OSA is 4 times greater than the influence of age and twice as great as the influence of male gender [44]. 

Obesity, macroangiopathy, hypertension, and dyslipidemia often coexist both in OSA and in NIDDM. However, factors other than obesity appear to play a significant role in the development of insulin resistance and metabolic disturbances in patients with OSA [45–50] including sleep fragmentation, increased sympathetic activity, and intermittent hypoxia [27, 51–54]. 

## 3. Obesity and Insulin Resistance

The prevalence of overweight and obesity in the United States and other industrialized countries is rapidly increasing. Over the last 40 years, the average body mass index (BMI) in men and women aged 20–74 yrs has increased from just over 25 Kg/M^2^ to almost 28 Kg/m^2^ [55–57]. 

It is also recognized that many subjects with OSA have central obesity and other features of metabolic syndrome [39], which is most widely accepted as being comprised of hyperinsulinemia, glucose intolerance, dyslipidemia, central obesity, and hypertension [58]. Insulin resistance is increased with increasing levels of obesity, but the reasons for this are not completely clear. As weight increases, the risk to develop complications such as hypertension, insulin resistance, diabetes mellitus, sleep disordered breathing, and obstructive sleep apnea syndrome increases [59]. 

The relationship of OSA with insulin resistance [47] may be the pathway that leads to increased risk for the development of cardiovascular disease in some patients. It has been observed that OSA patients have increased leptin [60] and C-reactive protein(CRP) [61], indicating a possible role in the pathogenesis of cardiovascular morbidity. Punjabi et al. [62] found that CRP is associated with nocturnal hypoxia. Thus, obesity is strongly associated with OSA [63], insulin resistance [64], leptin [65], and CRP [66] levels and may be the major confounding factor in the relationship of OSA to insulin resistance and cardiovascular morbidity. 

## 4. OSA and Insulin Resistance

Several studies have tried to establish the association between OSA and diabetes [67–70]. Van Cauter et al. have shown that experimentally induced acute sleep deprivation can cause a state of glucose intolerance [71]. OSA can affect the metabolism indirectly, by decreasing the quantity and/or quality of sleep [5]. Insulin resistance has been induced among healthy volunteers by sleep restriction. Sleep restriction on the other hand has also resulted in an increase in evening cortisol level and sympathetic activation [72]. 

A number of studies have also examined the cross-sectional relationship between OSA, as assessed by overnight polysomnography, and metabolic abnormalities [31, 39, 48–50, 67–69, 73–75]. Most of the studies suggest that OSA is related to impaired glucose tolerance and insulin resistance. Recent studies also confirm the high prevalence of habitual snoring in DM [73], or higher prevalence of metabolic syndrome in habitual snorers [74]. Over the past decade there has been increasing clinical and experimental evidence of the association between insulin resistance and OSA in non obese diabetic patients with autonomic neuropathy [75]. A laboratory-based investigation showed that diabetic patients with autonomic neuropathy are more likely to have OSA and central apnea than diabetic patients without autonomic neuropathy [76, 77]. 

Frequent snoring was associated with reduced glucose tolerance, as assessed by abnormal oral glucose tolerance tests (OGTTs) results and higher levels of HbA1c [78]. Another study done in the United States with a sample size of 150 healthy men reported that AHI and the degree of nocturnal desaturation were associated with glucose intolerance and insulin resistance independent of obesity [31]. 

Finally, concrete evidence came from the Sleep Heart Health Study [47]. In a community sample of 2656 subjects, the AHI and average oxygen saturation during sleep were associated with elevated fasting and 2-hour glucose levels during an oral glucose tolerance test. Sleep apnea severity was also associated with the degree of insulin resistance independent of BMI and waist circumference, amongst other confounders. 

Thus, there is a strong evidence which indicates that OSA and the risk of type 2 diabetes are associated, but the evidence supporting a role for OSA in the development of type 2 diabetes is still fairly limited. The reverse direction of causality (i.e., that diabetes may be a cause for breathing abnormalities during sleep) is also possible, as autonomic neuropathy could indeed disturb the control of respiration. 

Meslier et al. [79] studied 595 men who were referred to a sleep laboratory for suspected OSA. The cross-sectional data from polysomnography and 2-hour oral glucose tolerance tests (OGTTs) revealed that DM was present in 30.1% of OSA patients and 13.9% of nonapneic snorers. 

Studies in humans at high altitude [80] have indicated that sustained hypoxia adversely affects glucose tolerance and insulin sensitivity. Excessive daytime sleepiness (EDS) is a frequent, but not universal, symptom in patients with OSA [81] and recent evidence suggests that EDS may be an independent risk factor for diabetes [82]. In this study, it was hypothesized that EDS was associated with insulin resistance in OSA (independent of obesity), and that continuous positive airway pressure (CPAP) therapy improves both conditions, and our results confirm this hypothesis. The fact that EDS is also a marker of blood pressure response to CPAP therapy in patients with OSA [81–83] suggests that EDS is a potentially relevant clinical marker of several clinical manifestations of OSA. 

Vgontzas et al. [19] suggested that insulin resistance was a risk factor stronger than BMI and testosterone plasma levels for OSA and daytime sleepiness in premenopausal women suffering from polycystic ovarian syndrome. More recently, Punjabi et al. [84] found in 150 healthy mildly obese men that the severity of OSA correlated with levels of insulin 2 hours after an oral glucose load and reported a twofold increase in insulin resistance in subjects with an AHI 65, after controlling for BMI and percent body fat. Manzella et al. [75] observed that in 185 subjects with OSA, after adjusting for obesity, both AHI and minimum oxygen saturation were independent determinants of insulin resistance (the degree of insulin resistance increased by 0.5% for every single hourly increase in the AHI). 

**Figure 1:**
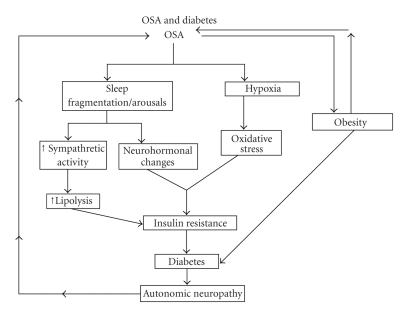
It illustrates the relationship of Obstructive Sleep Apnea and Diabetes.

Ficker et al. [85] assessed the presence of OSA (AHI 610) in a group of diabetic patients with and without diabetic autonomic neuropathy (DAN). They found a prevalence of Obstructive Sleep Apnea/Hypopnea Syndrome (OSAHS) amounting to 26% in diabetics with DAN; whereas none of the diabetics without DAN met the criteria for OSAHS. Neumann et al. [77] demonstrated a close correlation between nocturnal oxygen desaturation and the presence of DAN in a population of diabetic patients. In humans, as mentioned, several studies have shown increased sympathetic nervous system activity and increased catecholamine which in turn results in hyperinsulinemia [27, 72, 86–88]. Figure 1 illustrates the relationship between OSA, insulin resistance, and DM.

## 5. OSA and Endocrine

It is reported that the morbidity of acromegaly, diabetes, and thyroid disorders may, to a large extent, be ascribed to an altered sleep function [89]. 

Hypoxia mediated enhanced activation of the sympathoadrenergic system increasing plasma insulin despite the glycemic level was demonstrated in a study by Elmarsy et al. [48]. Sympathetic hyperactivity can influence glucose homeostasis by increasing glycogen breakdown and gluconeogenesis. Further, predisposition toward metabolic dysfunction in sleep apnea may also occur through its effects on the hypothalmic-pituitary-adrenal (HPA) axis. Experimental partial or total sleep deprivation has been shown to increase plasma cortisol level by 37% and 45%, respectively [90]. OSA to insulin resistance in humans has not been fully elucidated. Several plausible explanations can be proposed. Leproult et al. [91] hypothesized that the pathway between OSA and glucose intolerance was stimulation of the HPA axis due to hypoxias and fragmented sleep and leading to an increase in cortisol with corresponding hyperglycemia. For example, physical inactivity (due to day time somnolence) and sleep deprivation may be important contributing factors. OSA is also characterized by a proinflammatory state and elevated cytokine levels (e.g., tumor necrosis factor-alpha) which may lead to insulin resistance [52–54, 92]. TNF-*α* is usually elevated in individuals with obesity-induced insulin resistance. Studies have suggested that subjects with OSA had higher concentrations of IL-6 and TNF-*α* than obese subject without OSA [93]. 

Insulin resistance is also caused by increased lipolysis and fatty acid availability [93–95]. OSA may act through this mechanism by virtue of its association with central obesity and sympathetic activation [27]. Sympathetic activation rises circulating free fatty acids via stimulation of lipolysis and promotes insulin resistance [96]. 

Leptin, IL-6, and inflammatory mediators have also been implicated in the pathogenesis of insulin resistance and other features of metabolic syndrome [97, 98]. There have been several studies which have suggested an independent association of obstructive sleep apnea with several components of metabolic syndrome, particularly insulin resistance and abnormalities in lipid metabolism [14–17].

On the other hand, there is increasing evidence that Non Insulin Dependent Diabetes Mellitus (NIDDM) and OSA may be directly related throughout sleep disordered breathing-(SDB-) induced insulin resistance. Indeed, sleep fragmentation due to repetitive apneas may increase the plasma catecholamine and cortisol levels. These counter regulatory hormones induce glycogenolysis, gluconeogenesis, lipolysis with increased free fatty acid portal levels, and glucagon secretion, thus predisposing to hyperinsulinemia. However, other factors, including hypercarbia and recurrent arousals from sleep, can also increase autonomic output [27, 99, 100]. 

## 6. Diabetes and REM Sleep

Blood glucose homeostasis is subject to tight control exerted by the endocrine system [101]; nonetheless, both ultradian factors and different stages of sleep influence insulin secretion, concentration, and resistance [102, 103]. 

Insulin resistance increases towards the middle of the night with a subsequent decrease; as nonrapid eye movement (NREM) sleep is more frequent and longer in the first half of the night and rapid eye movement (REM) sleep in the second half of the night, sleep patterns may be implicated [104]. REM sleep is a physiologic and repetitive behavioral state in which high cerebral energy requirements correspond to a sustained neuronal activity [105]. REM sleep is accompanied by increased cerebral glucose utilization and cerebral blood flow [106, 107]. A decreased concentration of insulin and glucagon has been observed in REM sleep [102]. 

The higher prevalence of diabetes in OSA patients who have worse REM related respiratory events may be related to the unique neuroendocrine aspects of REM sleep, and their possible disruption as a consequence of sleep-disordered breathing. A decreased concentration of insulin and glucagon has been observed in REM sleep [108]. Hypoxemia is an important stimulus for altering autonomic activity, with larger desaturation causing greater increase in sympathetic activity [109, 110]. REM sleep is known to be associated with increased sympathetic activity [111]. REM sleep (and loss of muscle tone) triggers marked increases in sympathetic-nerve activity involving muscle blood vessels. REM-sleep twitches result in surges in blood pressure, and despite evidence of increased vasoconstriction in animals, we found a suppression of sympathetic-nerve activity in our subjects [111]. Thus, REM sleep is found to be associated not only with insulin resistance and diabetes but it also results in hypoxaemia due to sympathetic hyperactivity as well as a variable blood pressure surge which eventually increases the severity of diabetes and its risks. The study by Surani et al. [112] showed a very high prevalence of diabetes in an unselected cohort of Hispanic patients with obstructive sleep apnea compared to Caucasian. A REM apnea-hypopnea index of >20 was significantly associated with an increase prevalence of diabetes in Hispanic population. The brain, which constitutes 2% of body mass, depends entirely on glucose metabolism and utilizes approximately 50% of total body glucose [113]; it extracts about 10% of blood glucose without the need of insulin to cross the blood-brain barrier due to facilitated glucose transport [114]. In numerous physiological respects, REM is a distinctive sleep phase, with brain activation reflected by a 30% larger blood flow compared with quiet wakefulness, which relates to augmented glucose consumption [115]. 

## 7. OSA Treatment (CPAP) and Insulin Resistance

The effect of CPAP treatment on glucose metabolism has been evaluated in multiple studies [93, 116–129]. Trenell et al. reported that CPAP treatment for 2 days rapidly improved the insulin sensitivity in nondiabetic patients and the effect of CPAP persisted for approximately 3 months after treatment [123]. 

Brooks et al. found that obese patients with DM often complained of excessive daytime sleepiness, fatigue, and tiredness. In their study, patients with OSA were treated with CPAP. After four months of treatment, the insulin responsiveness had significantly improved [130]. Several other studies about successful treatment of OSA with CPAP have been shown to produce improvement in insulin sensitivity [59, 110, 119, 121, 122, 130–132]. Interestingly, most of the studies suggest that the lower the BMI, the better response in the insulin sensitivity improvement after CPAP treatment [59]. When OSA is timely and properly treated the results seen are not only a decrease in daytime excessive sleepiness, but also a decrease in cardiovascular risk, and in improvement of insulin resistance [1, 22]. In a study by Babu et al. [122], subjects who used CPAP for more than four hours per day had a significant reduction in HbA1c level. Recent studies have linked untreated sleep disordered breathing to hypertension, insulin resistance, coronary disease, congestive heart failure, stroke, obesity, and gastro esophageal reflux [59]. 

More recently, in a population-based sample, Lindberg et al. [133] showed reductions in fasting insulin levels and insulin resistance (estimated by HOMA) after 3 weeks of CPAP treatment in 28 men with OSA compared with matched nonapneic (AHI, _ 10) control subjects followed over the same time period without CPAP therapy. Czupryniak et al. [121] also suggested that in non-diabetic patients, increased blood glucose was seen after one night of CPAP therapy, with a tendency to higher fasting insulin and insulin resistance after CPAP. This was felt to be secondary to a CPAP-related increase in growth hormone. A few studies reported decrease in visceral fat after CPAP use [131] while another study found no change [134]. Three independent preliminary studies presented in abstract form have suggested a positive response to CPAP therapy with improvements in insulin sensitivity [135], fasting, [136], and nocturnal [137] glucose levels in both diabetic and nondiabetic patients with OSA. However, the effect of CPAP therapy on metabolic syndrome is controversial. The author feels that recent studies have increasingly been favoring the role of CPAP therapy in enhancing insulin sensitivity. Several studies are currently ongoing which we hope can help to resolve the issue.

## 8. Conclusion

Diabetes Mellitus and Obstructive Sleep Apnea are extremely common medical conditions that are prevalent in our population. There is mounting evidence which links sleep deprivation, obesity, and sleep-related breathing to diabetes. Studies have established the association of OSA with diabetes as well as the importance of timely CPAP therapy in decreasing the insulin resistance in patients. However, despite the availability of convincing evidence and abundant cross-sectional studies, there is clearly a substantial requirement for a well-designed prospective study to clearly address these issues in depth. It is imperative for physicians to have a high degree of suspicion regarding the reciprocal prevalence of diabetes mellitus and obstructive sleep apnea. Moreover, there is lot to be done in educating Family physicians regarding the association of OSA and diabetes.
